# Dimensions rénales en Service de Néphrologie Clinique, Befelatanana, Antananarivo

**DOI:** 10.11604/pamj.2016.24.117.9411

**Published:** 2016-06-06

**Authors:** Benja Ramilitiana, Mihary Dodo, Henintsoa Nirina Rakotoarimanga, Rado Lalao Randriamboavonjy, Willy Franck Randriamarotia

**Affiliations:** 1 Service de Néphrologie-Dialyse, Centre Hospitalier Universitaire Joseph Raseta Befelatanana, Antananarivo, Madagascar; 2 Laboratoire LARTIC, faculté de Médecine, Antananarivo, Madagascar

**Keywords:** Dimensions, échographie, reins, Dimensions, ultrasound, kidneys

## Abstract

La connaissance des dimensions rénales pour tout individu et pour toute ethnie, aide à une décision médicale d'ordre diagnostique et thérapeutique. Pour un pays à faible revenu comme Madagascar, l’échographie constitue un outil idéal à cette fin. Ce travail a comme objectifs de recueillir les dimensions rénales, puis de chercher la corrélation entre ces dernières et les paramètres démographiques ou anthropométriques des Malgaches. Il s'agit d'une étude rétrospective, s’étalant sur une période de 3 ans, effectuée dans le service de Néphrologie du centre hospitalier de Befelatanana, Antananarivo, recrutant 200 patients non diabétiques, sans maladie rénale chronique. L’âge moyen de nos patients était de 45 ans ±16, avec un sex ratio de 0,9. En moyenne, les dimensions (longueur × largeur × épaisseur) étaient de 98 mm × 42 mm × 30 mm pour le rein droit et 99 mm × 45 mm × 31 mm pour le rein gauche. Nous avons retrouvé une différence significative entre le rein droit et gauche sur la longueur (p<0,00001) et la largeur (p = 0,03). Une relation significative était aussi retrouvée entre la longueur rénale et l’âge (p = 0,0016 et p = 0,04 respectivement pour le rein droit et le rein gauche). Aucune relation significative n'a été établie entre les dimensions rénales et la taille ni le poids de nos patients. Malgré ses limites, notre étude apporterait une aide sur le plan pratique clinique ainsi que pour une étude ultérieure.

## Abstract (en)

The knowledge of normale renal sizes specific for each individual and for any ethnicity, allows diagnostic and therapeutic medical decision. For a low-income country such as Madagascar, ultrasound is an ideal tool for this purpose. This study aims to collect data on renal dimensions and to seek the correlation between these data and the demographic and anthropometric parameters in Malagasies. This is a retrospective study of 200 non-diabetic patients without chronic kidney disease, spanning 3 years, conducted in the Nephrology Department of Befelatanana Hospital, Antananarivo. The average age of our patients was 45 ±16 years, with a sex-ratio of 0,9. On average, the dimensions (length x width x thickness) were 98 mm × 42 mm × 30 mm for right kidney, 99 mm × 45 mm × 31 mm for the left kidney. We found a significant difference between the right and left kidney in length (p<0.00001) and width (p = 0.03). A significant relationship was also found between renal length and age (p = 0.0016 and p = 0.04 respectively for the right kidney and the left kidney). No significant relationship was found between renal dimensions and size and patients weight. Despite its limitations, our study would provide important supports for clinical practice as well as for further studies.

## Aux éditeurs du Journal Panafricain de Médecine

Dans un pays à faible revenu comme Madagascar, l’échographie est un moyen pas coûteux, non invasif, largement disponible et simple en imagerie rénale. Cette exploration tient une place non négligeable pour chercher diverses anomalies en pratique clinique. Mais peu d’études étaient effectuées à Madagascar concernant les dimensions rénales [1–4], alors que la connaissance des valeurs habituelles chez les malgaches aide à une décision médicale particulière, entre autre la prescription pour la biopsie rénale ou l'initiation d'un traitement spécifique. Les objectifs de ce travail étant de recueillir les dimensions des reins à partir des dossiers d'un service clinique, de rapporter la corrélation entre ces dimensions et les paramètres démographique et anthropométrique des patients malgaches. Notre étude, rétrospective, réalisée au service de Néphrologie de l'hôpital Joseph Raseta de Befelatanana, Antananarivo, contient 200 patients non diabétiques, sans maladie rénale chronique, recrutés entre janvier 2010 et décembre 2013. Les patients inclus avaient une échographie sans anomalie rénale ni des voies urinaires, un débit de filtration glomérulaire estimé normal. Le débit de filtration glomérulaire était estimé par la formule MDRD simplifiée. L’échographie était effectuée à Antananarivo. La créatininémie était dosée dans différents laboratoires à Antananarivo. Les dimensions rénales, les paramètres démographique et anthropométrique étaient recueillis dans les dossiers d'hospitalisation des patients inclus. L’étude des corrélations entre les dimensions rénales et le genre, l′âge, la taille, le poids était effectué dans ce travail. Les données étaient exprimées sous forme de moyenne avec ecart-type. Les données de base étaient collectées en utilisant le logiciel Excel (Microsoft). Le test statistique était fait en utilisant le logiciel R. L'analyse est significative quand p< 0,05. Sur ces 4 ans d’étude, 200 patients étaient colligés, avec 95 hommes (47,5%) et 105 femmes (52,5%) donnant un sex-ratio de 0,9. L’âge médian de notre population d’étude était de 45 ans (17 à 91 ans). Le Tableau 1 rapporte les caractéristiques démographique et biométrique des patients inclus. Le rein droit avait une dimension légèrement plus petite que le rein gauche, soit sur la longueur (p<0,00001), ou sur la largeur (p = 0,03) mais la différence n’était pas significative sur l’épaisseur (p = 0,06). Pour le rein droit, la longueur rénale avait une relation positive avec l’âge (p = 0,0016), la largeur avec le poids du sujet (p = 0,032). Pour le rein gauche, la longueur rénale avait une relation positive avec l’âge (p = 0,04), elle avait aussi un lien significatif avec le poids du sujet (p = 0,03499). L’épaisseur des reins n'avait pas de relation significative avec la biométrie du sujet.

**Tableau 1:** Caractéristiques des patients inclus

Paramètres	Moyen ± écart-type	Moyen ± écart-type	Moyen ± écart-type
Genre(n)	Total(200)	homme(95)	femme(105)
Age (ans)	45 ±16	49 ±16	42 ±15
Poids moyen (kg)	52 ± 10	54 ± 9	50 ± 10
Taille moyenne (cm)	160±8	165±7	156±7
Surface corporelle (m^2^)	1,53 ±0,15	1,58 ±0,14	1,5 ±0,15
Indice de masse corporelle (kg/m^2^)	20± 3,8	19± 3	21± 4
Créatininémie (µmol/L)	87±19,6	-	-
Débit de filtration glomérulaire (ml/mn)	95± 37,2	-	-
Rein droit			
longueur (mm)	98±10	98±8	99±10
largeur (mm)	42±7	43±7	42±7
épaisseur (mm)	30± 16	32± 17	28± 15
Rein gauche			
longueur (mm)	99±10	100±10	99±10
largeur (mm)	45±6	45±8	45±7
épaisseur (mm)	31± 16	33± 18	30± 14

Notre étude rapporte les valeurs biométriques des reins d'un adulte malgache, issues des résultats d'un service clinique en se basant sur une exploration échographique, éléments essentiels pour aider un clinicien à prescrire un acte diagnostique ou thérapeutique: réaliser une biopsie rénale ou argumenter la sévérité d'une insuffisance rénale. Ces décisions se basent surtout sur la longueur rénale qui est un bon indicateur de la dimension rénale en pratique clinique [5, 6]. Pour Ahmad et al, l’étude effectuée chez des malgaches adultes rapportait les dimensions suivant: le rein droit mesurait 91,16 mm de hauteur, 48,18 mm de largeur et 37,04 mm d′épaisseur. Le rein gauche faisait 90,6 mm de hauteur, 37,04 mm de largeur, 40,4 mm d′épaisseur. L′âge moyen pour la série d'Ahmad et al [1] était plus jeune avec un médian à 36,83 ans (18 à 77 ans) par rapport aux patients dans notre série, expliquant la tendance plus inférieure des différentes mesures dans leur série. L’âge a une corrélation positive avec la dimension du rein, mais qui s'inverse ensuite après 60 ans [7]. Dans notre série et dans les séries antérieures [1, 7], le rein gauche avait une dimension plus grande que le rein droit; cette différence entre les 2 reins serait due au fait que le foie est plus grand que la rate et le rein droit a moins d'espace pour son développement [7]; l'artère rénale gauche est moins courte avec comme conséquence un flux de sang plus important pour le rein gauche [7]; enfin, la taille plus petite des patients limiterait le développement longitudinal des 2 reins [7]. Dans notre série, aucune corrélation entre le genre et les dimensions rénales était constatée statistiquement, même si la série de Glodny et al rapportait que le rein de l'homme est plus grand que ce de la femme, par l'influence de l'hormone sexuelle sur cette corrélation [8]. Dans notre résultat et celui d'une étude antérieure chez les tananariviens adultes [1], aucune relation n’était constatée entre la taille du patient et les dimensions des reins. Les limites de notre étude sont non négligeables, les résultats des échographies étaient recueillis rétrospectivement à partir des dossiers des patients d'un service clinique. Ces échographies étaient issues de différents centres d’échographie d'Antananarivo, faites par différents opérateurs avec différents appareils d’échographie, les résultats étant opérateur-dépendants; même si la mesure de la longueur rénale varie moins par rapport à la mesure du volume rénal [5, 6].

## Conclusion

Les données issues de notre étude vont servir de bases en se rajoutant à celles obtenues antérieurement chez la population malgache, en attendant les études sur de large échantillon et les méta-analyses.
